# Predictors of target lesion revascularization after paclitaxel-coated balloon angioplasty for *de novo* coronary artery lesions

**DOI:** 10.3389/fcvm.2026.1724573

**Published:** 2026-03-24

**Authors:** Tommy Suharjo, Aninka Saboe, Dendi Puji Wahyudi, Achmad Fauzi Yahya

**Affiliations:** Department of Cardiology and Vascular Medicine, Faculty of Medicine, Universitas Padjadjaran—Dr. Hasan Sadikin General Hospital, Bandung, Indonesia

**Keywords:** calcified lesion, *de novo* coronary artery disease, drug-coated balloon (DCB), percutaneous coronary intervention, target lesion revascularization

## Abstract

**Background:**

Evidence is limited on factors driving target lesion revascularization (TLR) after drug-coated balloon (DCB) treatment for *de novo* coronary lesions. This study evaluated the incidence and determinants of TLR following paclitaxel-coated balloon angioplasty in an Indonesian population.

**Methods:**

Single-center retrospective analysis of a prospective cohort PCI registry study included 112 patients (129 lesions) who underwent successful DCB angioplasty in *de novo* coronary lesions between January 2020 and December 2024 and follow-up angiography. The primary endpoint was incidence and predictors of TLR. Secondary endpoints included all-cause mortality and major adverse cardiovascular events (MACE) at 12 months.

**Results:**

Over a median angiographic evaluation of 139 days (IQR, 100—291 days), TLR events occurred in 16 lesions (12.4%) and were associated with calcified lesions (adjusted OR 9.93; 95% CI, 1.23–80.46; *p* = 0.032. Baseline clinical characteristics, reference vessel diameter, intravascular imaging utilization, predilation procedure, balloon inflation time, maximal inflation pressure, and the presence of non–flow-limiting dissection were not associated with TLR. At 12 months, MACE (a composite of cardiac death, recurrent myocardial infarction, target lesion revascularization, unplanned rehospitalization, or cerebrovascular accident) occurred in 14 patients (12.5%) with no all-cause or cardiac mortality observed.

**Conclusion:**

Calcified coronary lesions independently predicted TLR after paclitaxel-coated balloon angioplasty for *de novo* coronary artery lesions.

## 1 Introduction

In patients with obstructive coronary artery disease undergoing percutaneous coronary intervention (PCI), drug-eluting stents (DES) substantially reduce the risk of restenosis and stent thrombosis (1, 2). Nevertheless, persistent concerns regarding late stent thrombosis and restenosis have prompted the development of DCB therapy as a stentless revascularization strategy designed to avoid long-term stent-related complications (3).

DCB refers to angioplasty balloons coated with antiproliferative drug, designed to deliver the drug to the vascular wall during balloon inflation (4). The rationale behind DCB technology is to combine mechanical luminal expansion with local drug delivery, enabling treatment of coronary lesions without permanent metallic scaffolds, and thereby eliminating the risk of stent thrombosis and stent failure (3). The absence of permanent implant may also preserve options for future bypass surgery and allow for shorter durations of dual antiplatelet therapy, potentially reducing bleeding risk (2).

Current European and Japanese guidelines recommend DCB angioplasty for the treatment of in-stent restenosis in both DES and bare-metal stents with a Class IA endorsement, whereas its role in *de novo* coronary lesions remains less well established (4, 5). In the United States, one DCB is currently approved for the treatment of coronary in-stent restenosis (6).

Despite growing interest in DCB therapy for *de novo* coronary lesions, evidence on factors associated with TLR remains limited, particularly in real-world Southeast Asian population. Patients from Southeast Asia—a subgroup of the Asia-Pacific region—differ from Western populations in both demographics and coronary disease characteristics, commonly presenting with smaller vessel diameters and longer, diffuse lesions, features associated with higher rates of restenosis and repeat revascularization (7). This study aimed to determine the incidence and predictors of TLR following DCB treatment in patients with *de novo* coronary artery disease. We hypothesized that identifying lesion- and procedure-related factors linked to TLR on follow-up angiography could guide optimal lesion preparation and DCB implantation strategies. Accordingly, we performed a single-center, retrospective analysis of a prospective cohort to evaluate the incidence and determinants of TLR in patients undergoing DCB angioplasty for *de novo* coronary lesions.

## 2 Methods

### 2.1 Study design and population

This was a single-center, retrospective analysis of a prospective PCI registry of Dr. Hasan Sadikin General Hospital (Bandung, Indonesia). Between January 2020 and December 2024, 372 patients with 449 *de novo* coronary artery lesions underwent successful DCB angioplasty. Follow-up angiography was performed for scheduled evaluation, staged PCI, or unplanned PCI for acute coronary syndrome (ACS). Lesions were categorized into TLR and non-TLR groups.

Inclusion criteria comprised patients aged ≥ 18 years with *de novo* coronary artery lesions requiring revascularization for ACS or chronic coronary syndromes (CCS) who underwent successful DCB angioplasty [defined as patients with TIMI flow 3, absence of significant coronary dissection (type C-F) and residual stenosis ≤30%], subsequently underwent follow-up angiography, and completed clinical follow-up in 12 months.

Exclusion criteria were non-adherence to dual antiplatelet therapy after DCB angioplasty, severe primary valvular disease, non-ischemic cardiomyopathy, congenital heart disease, declined angiography follow up, bailout stenting, and lost to follow-up during major adverse cardiovascular events (MACE) evaluation. In addition, patients with an indeterminate DCB site on follow-up angiography because of the possibility of stent failure proximal to the DCB segment area were also excluded.

The study complied with the Declaration of Helsinki and was approved by the Ethics Committee of Dr. Hasan Sadikin General Hospital, Bandung, West Java, Indonesia (DP.04.03/XIV.6.5/17/2024).

### 2.2 Interventional procedure and devices

The selection of vascular access, guide catheter, guidewire, balloon type, PCI strategy (DCB-only or hybrid with DES), use of intravascular imaging (IVI), and administration of glycoprotein IIb/IIIa receptor inhibitors were left to operator discretion. Contemporary balloon angioplasty using semi compliant balloon, scoring balloon, or cutting balloon was performed in all *de novo* lesions to achieve a stenosis of ≤30%, with a recommended balloon-to-vessel ratio of 0.8–1.0. Patients who developed a flow-limiting dissection after predilation or exhibited a type D–F coronary dissection underwent bailout DES implantation and were subsequently excluded from the analysis. The intervention procedure was performed according to the international and Asia-Pacific consensus recommendations for DCB treatment.

Intravascular imaging was performed in selected lesions using optical coherence tomography (OCT; Dragonfly OPTIS, Abbott Vascular), intravascular ultrasonography (IVUS; Opticross, Boston Scientific) or both. When utilized, IVI was conducted prior to predilation. If the imaging catheter could not cross the lesion, predilation with a 2.0 mm semi-compliant balloon (SCB) was performed before catheter advancement. In the IVI group, lesion preparation and DCB sizing were tailored based on morphological assessment and reference vessel size measurements obtained from IVI.

Paclitaxel-coated balloons used including SeQuent Please (B. Braun, Melsungen, Germany), Swide (Shenqi Medical Technology, China), and AGENT monorail (Boston Scientific, USA). Number of DCB applied per lesion was recorded.

All patients received standard dual antiplatelet therapy (DAPT) with aspirin and P2Y12 inhibitors (clopidogrel or ticagrelor) before the procedure. DAPT was continued for at least six months in CCS treated with DCB only approach, and 12 months in ACS or CCS treated with hybrid approach involving DES implantation.

### 2.3 Angiographic and intravascular imaging data

The target lesion was defined as the treated segment including the 5-mm margin proximal and distal to the DCB balloon. Lesion length was determined using the length of the DCB, or when multiple devices were deployed, as the sum of the DCB and DES lengths. Calcified lesions were identified angiographically by presence of radiopaque densities noted during or without cardiac motion involving one or both sides of the vascular wall. For lesions evaluated with intravascular imaging, calcified lesions were identified on IVUS as bright echoes with acoustic shadowing, whereas on OCT it appeared as a heterogeneous area of low backscatter with low attenuation and clear borders (8). Validated intravascular imaging–based calcium scoring systems include the OCT-derived score by Fujino et al., which incorporates maximum calcium arc ≥180°, thickness ≥0.5 mm, and calcium length ≥5 mm, and the IVUS calcium scoring systems described by Zhang et al., which assigns one point each for superficial calcium angle ≥270° in ≥5 mm length, 360° of superficial calcium, calcified nodule, and vessel diameter <3.5 mm (score range 0–4) (9, 10). Calcified lesions were categorized as severe or non-severe. Severe calcification was defined angiographically as radiopaque densities noted without cardiac motion generally involving both sides of the arterial wall; when intravascular imaging was available, an OCT calcium score ≥2 or an IVUS calcium score ≥2 was classified as severe. Quantitative vessel measurements included the proximal and distal reference diameter, external elastic lamina (EEL), or external elastic membrane (EEM) at the target lesion obtained by OCT or IVUS in patients who undergoing intravascular imaging. In the non–imaging-guided group, vessel size was estimated angiographically, based on visual assessment of the reference segment in at least two orthogonal projections, and the selected optimal DCB diameter was documented.

### 2.4 Endpoints

The primary endpoint was TLR, defined as repeat percutaneous intervention of the target lesion or bypass surgery of the target vessel performed for restenosis or other complication of the target lesion. Secondary endpoints included all-cause and cardiac mortality, and major adverse cardiovascular events (MACE—a composite of cardiac death, recurrent myocardial infarction, target lesion revascularization, unplanned rehospitalization, or cerebrovascular accident) during at least 12 months of follow-up. Clinical follow up data were obtained at outpatient clinical visits or telephone interviews. All angiographic studies pertaining to TLR were independently reviewed by a panel of interventional cardiologists blinded to clinical outcomes. MACE was assessed on a patient basis, while TLR were evaluated on a lesion basis.

### 2.5 Statistical analysis

Statistical analyses were performed using SPSS, version 24.0 (SPSS Inc., Chicago, IL). Continuous variables are summarized as mean ± SD or median (IQR), as appropriate, and categorical variables as counts (percentages). Baseline clinical, angiographic, and procedural characteristics were compared between TLR and non-TLR groups. Categorical variables were analyzed using the *χ*² test or Fisher's exact test. Continuous variables were analyzed with the independent-samples *t* test or the Mann–Whitney *U* test, as appropriate. A two-sided *p* ≤ 0.05 was considered statistically significant.

Variables with *p* < 0.15 on univariable analysis were entered into a multivariable logistic regression model to identify independent predictors of TLR. Adjusted odds ratios (ORs) with 95% confidence intervals (CIs) were reported. Time-to-event analyses for TLR and MACE were performed using the Kaplan–Meier method, with group comparisons conducted using the log-rank (Mantel–Cox) test.

Multicollinearity among candidate predictors was assessed using variance inflation factors (VIF) and condition indices. VIF >5 and condition index >30 were considered indicative of significant collinearity.

## 3 Results

### 3.1 Baseline characteristics

**Figure 1:**
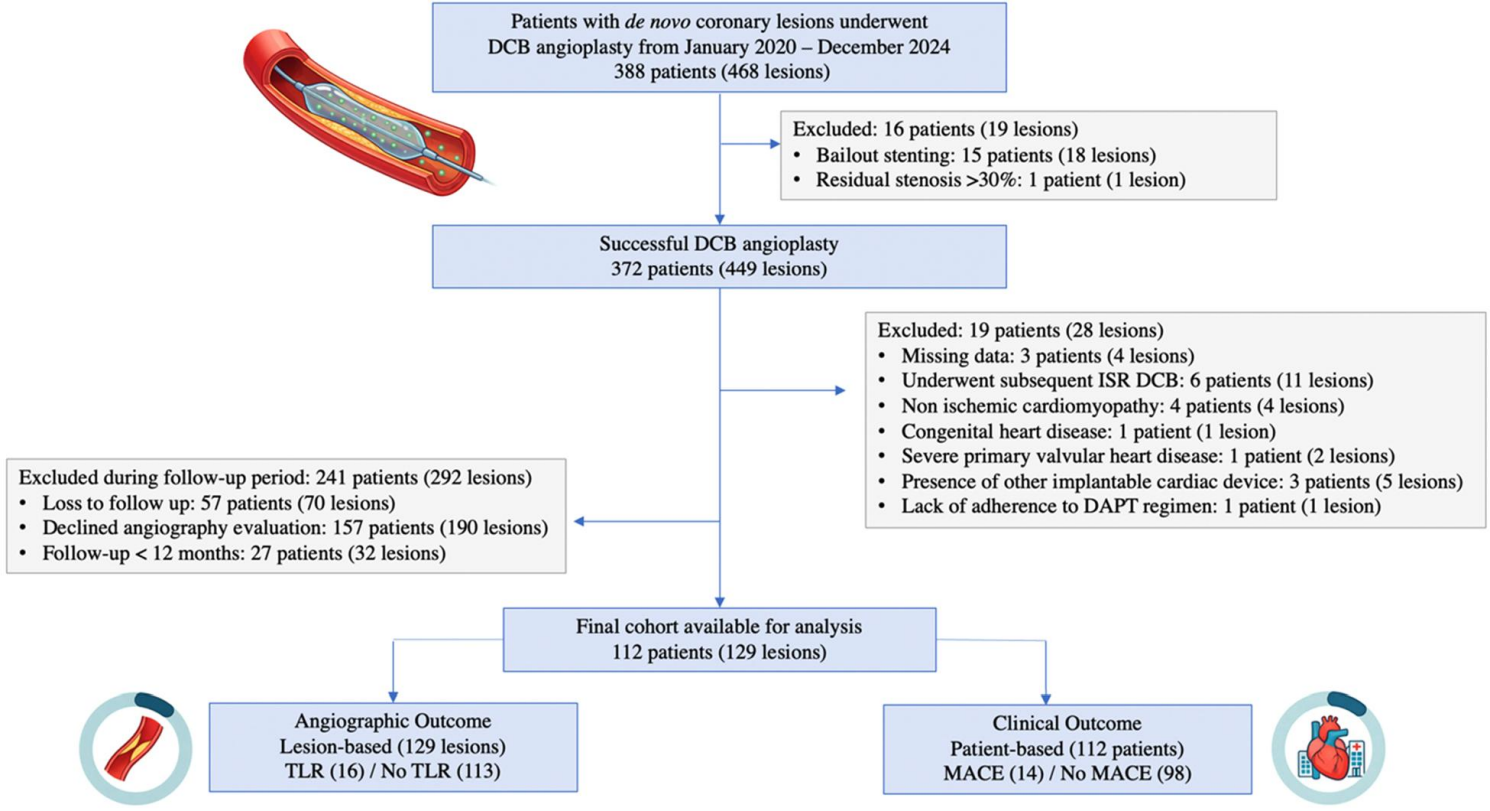
Study flowchart.

A total of 129 lesions treated with DCB angioplasty and underwent angiographic evaluation were analyzed (Figure 1). Sixteen lesions (12.4%) developed TLR during follow-up. The mean patient age was 59.9 ± 9.1 years, and most patients were male (76.3%). The median interval to follow-up angiography was 139 days (IQR, 100–291 days), with no significant difference between the TLR and non-TLR groups. Smoking was more prevalent among patients with TLR (81.3% vs. 54.0%, *p* = 0.04), however this association did not persist after adjustment in the multivariable model. Other baseline characteristics, including diabetes mellitus, hypertension, dyslipidemia, prior myocardial infarction, prior stroke, and history of previous PCI did not differ significantly between groups. Notably, approximately one-quarter of the study population had heart failure with reduced ejection fraction (HFrEF). Detailed baseline characteristics are summarized in Table 1.

**Table 1:** Baseline characteristics.

Variable	All Lesions	TLR	Non-TLR	*p*-value
	(*n* = 129)	(*n* = 16)	(*n* = 113)	
Angiographic evaluation, median (Q1-Q3)	139 (100–291)	202 (126–294)	134 (99–272)	0.721
Age (years)	59.9 ± 9.1	58.7 ± 8.8	60.0 ± 9.2	0.534
Sex, *n* (%)				
Male	99 (76.3%)	15 (93.78%)	84 (74.3%)	0.116
Female	30 (23.3%)	1 (6.3%)	29 (25.7%)	
Diabetes Mellitus, *n* (%)	35 (27.1%)	2 (12.5%)	33 (29.2%)	0.232
Hypertension, *n* (%)	74 (57.4%)	11 (68.8%)	63 (55.8%)	0.325
Smoking, *n* (%)	74 (57.4%)	13 (81.3%)	61 (54.0%)	0.039*
Dyslipidemia, *n* (%)	55 (42.6%)	9 (56.3%)	46 (40.7%)	0.239
Family History of CAD, *n* (%)	13 (10.1%)	1 (6.3%)	12 (10.6%)	1.000
Renal Failure, *n* (%)	3 (2.3%)	0 (0%)	3 (2.7%)	1.000
LVEF %	50.2 ± 13.4	47.8 ± 12.9	50.5 ± 13.5	0.521
LVEF < 40%, *n* (%)	33 (25.8%)	5 (31.3%)	28 (25.0%)	0.970
History of MI, *n* (%)	57 (44.2%)	7 (43.6%)	50 (44.2%)	0.785
Previous PCI, *n* (%)	42 (32.8%)	3 (20.0%)	39 (34.5%)	0.382
Previous Stroke, *n* (%)	5 (3.9%)	1 (6.3%)	4 (3.5%)	0.490

Categorical variables are presented as number (%) and were analyzed using the chi-square test or Fisher's exact test, as appropriate. Numerical variables with a non-normal distribution are presented as median (interquartile range) and were analyzed using the Mann–Whitney *U* test. Numerical variables with a normal distribution are presented as mean ± standard deviation and were analyzed using the independent *t*-test. *P*-values represent comparisons between the TLR and non-TLR groups for baseline characteristics. CAD, coronary artery disease; LVEF, left ventricular ejection fraction; MI, myocardial infarction; PCI, percutaneous coronary intervention; TLR, target lesion revascularization.

* Indicates statistical significance (*p* value ≤ 0.05).

### 3.2 Angiographic, intravascular imaging, and PCI findings

Baseline angiographic and procedural characteristics are summarized in Tables 2, 3. DCB charactertistics are presented in Tables 4. The predominant clinical presentation was chronic coronary syndrome, most often treated with a planned PCI strategy. The mean reference vessel diameter was 2.88 ± 0.46 mm, and 69.8% of lesions involved non-small vessels (≥2.75 mm), with a mean DCB-to-reference diameter ratio of 0.95 ± 0.11. The left anterior descending artery (LAD) represented the most common target vessel (48.8%), followed by the left circumflex (27.1%) and right coronary arteries (17.8%). At the coronary segment level, more than half of treated lesions spanned multiple coronary segments (56.6%). Detailed lesion anatomical distribution is summarized in Table 3. Lesion subsets also included left main (3.1%), bifurcation (20.2%), ostial (29.5%), chronic total occlusion (12.4%), and long diffuse (7.8%) lesions.

**Table 2:** Procedural characteristics.

Variable	All Lesions	TLR	Non-TLR	*p*-value
	(*n* = 129)	(*n* = 16)	(*n* = 113)	
Clinical presentation at initial procedure, *n* (%)				0.355
Chronic coronary syndrome	98 (76.0%)	14 (87.5%)	84 (74.3%)	
Acute coronary syndrome	31 (24.0%)	2 (12.5%)	29 (25.7%)	
Reason for angiographic evaluation, *n* (%)				0.401
Acute coronary syndromes	6 (5.0%)	0 (0.0%)	6 (5.6%)	
Staging or chronic coronary syndromes	41 (35.4%)	7 (52.6%)	34 (33.1%)	
Angiography evaluation	82 (59.6%)	9 (47.4%)	73 (61.3%)	
PCI Strategy				0.667
Planned/Staged	108 (83.7%)	15 (93.8%)	93 (82.3%)	
Primary	5 (3.9%)	0 (0.0%)	5 (4.4%)	
Rescue	2 (1.6%)	0 (0.0%)	2 (1.8%)	
Inpatient	14 (10.9%)	1 (6.3%)	13 (11.5%)	
Access site, *n* (%)				0.623
Radial	73 (57.0%)	9 (56.3%)	64 (57.1%)	
LDTR	6 (4.7%)	0 (0.0%)	6 (5.4%)	
Femoral	29 (22.5%)	5 (31.3%)	24 (21.2%)	
Double access	21 (16.4%)	2 (12,5%)	19 (17.0%)	
IABP	7 (5.4%)	0 (0.0%)	7 (6.2%)	0.596

*p*-value represents comparison between TLR and non-TLR groups. IABP, intra aortic balloon pump; LDTR, left distal trans radial; PCI, percutaneous coronary intervention; TLR, target lesion revascularization.

**Table 3:** Lesion characteristics and preparation.

Variable	All Lesions	TLR	Non-TLR	*p*-value
	(*n* = 129)	(*n* = 16)	(*n* = 113)	
Lesion Characteristics				
Number of diseased vessels, *n* (%)				0.991
1 vessel disease	16 (12.4%)	2 (12.5%)	14 (12.4%)	
2 vessel disease	34 (26.4%)	4 (25.0%)	30 (26.5%)	
3 vessel disease	79 (61.2%)	10 (62.5%)	69 (61.1%)	
Multivessel disease (≥2 VD), *n* (%)	113 (87.6%)	14 (87.5%)	99 (87.6%)	1.000
Reference vessel diameter (mm), *n* (%)	2.88 ± 0.46	2.81 ± 0.40	2.89 ± 0.47	0.552
Non-small vessel (≥2,75 mm), *n* (%)	90 (69.8%)	11 (68.8%)	79 (69.9%)	1.000
Target coronary lesion (DCB), *n* (%)				0.728
LAD	63 (48.8%)	7 (43.8%)	56 (49.6%)	
LCx	35 (27.1%)	4 (25.0%)	31 (27.4%)	
RCA	23 (17.8%)	3 (18.8%)	20 (17.7%)	
LMCA	4 (3.1%)	2 (12.5%)	2 (1.8%)	
RI	4 (3.5%)	0 (0.0%)	4 (3.5%)	
Coronary artery segments, *n* (%)				0.217
Proximal	24 (18.6%)	7 (43.8%)	17 (15.0%)	
Mid	11 (8.5%)	1 (6.3%)	10 (8.8%)	
Distal	15 (11.6%)	1 (6.3%)	14 (12.4%)	
Proximal to mid	9 (7.0%)	0 (0.0%)	9 (8.0%)	
Proximal to distal	14 (10.9%)	3 (18.8%)	11 (9.7%)	
Mid to distal	18 (14.0%)	2 (12.5%)	16 (14.2%)	
Ostial	6 (4.7%)	0 (0.0%)	6 (5.3%)	
Ostial to Proximal	20 (15.5%)	1 (6.3%)	19 (16.8%)	
Ostial to mid	6 (4.7%)	1 (6.3%)	5 (4.4%)	
Ostial to distal	6 (4.7%)	0 (0.0%)	6 (5.3%)	
Ostial lesion, *n* (%)	38 (29.5%)	4 (25.0%)	34 (30.1%)	0.777
Calcified lesion, *n* (%)	77 (59.7%)	15 (93.8%)	62 (54.9%)	0.003*
CTO, *n* (%)	16 (12.4%)	2 (12.5%)	14 (12.4%)	1.000
Bifurcation lesion, *n* (%)	26 (20.2%)	3 (18.8%)	23 (20.4%)	1.000
Lesion Preparation				
Use of IVI, *n* (%)	104 (80.6%)	14 (87.5%)	90 (79.6%)	0.736
Type of IVI, *n* (%)				0.826
None	25 (20.4%)	2 (12.5%)	23 (20.4%)	
IVUS	88 (68.2%)	12 (75.0%)	76 (67.3%)	
OCT	14 (10.9%)	2 (12.5%)	12 (10.6%)	
IVUS + OCT	2 (1.6%)	0 (0%)	2 (1.8%)	
Predilation procedure				
Predilation device diameter (mm)	2.68 ± 2.04	2.47 ± 0.55	2.71 ± 2.17	0.625
Predilation device length (mm)	14.0 ± 2.32	14.6 ± 2.70	13.9 ± 2.26	0.479
Atherectomy, *n* (%)				0.057
No	112 (86.8%)	13 (81.3%)	99 (87.6%)	
Rotational	8 (6.2%)	3 (18.8%)	5 (4.4%)	
Orbital	9 (7.0%)	0 (0.0%)	9 (8.0%)	
Preparation Balloon				
Semi-compliant balloon	61 (47.3%)	8 (50.0%)	53 (46.9%)	0.816
Cutting balloon	48 (37.2%)	6 (37.5%)	42 (37.2%)	0.979
Non-slip element balloon	34 (26.4%)	6 (37.5%)	28 (24.8%)	0.280
Non-compliant balloon	55 (42.6%)	7 (43.8%)	48 (42.5%)	0.923

*p*-value represents comparison between TLR and non-TLR groups. CTO, chronic total occlusion; LAD, left artery descending; LDTR, left distal trans radial; LCx, left circumflex artery; LMCA, left main coronary artery; RCA, right coronary artery; RI, ramus intermedius; IVI, intravascular imaging; IVUS, intravascular ultrasound; OCT, optical coherence tomography; PCI, percutaneous coronary intervention; TLR, target lesion revascularization.

* indicates statistical significance (*p* value ≤ 0.05).

**Table 4:** DCB characteristics.

Variable	All Lesions	TLR	Non-TLR	*p*-value
	(*n* = 129)	(*n* = 16)	(*n* = 113)	
DCB-only or Hybrid, *n* (%)				0.053
DCB-only	52 (40.3%)	10 (62.5%)	42 (37.2%)	
Hybrid DES/DCB	77 (59.7%)	6 (37.5%)	71 (62.8%)	
Type of DCB used				0.108
SeQuent Please	111 (86.0%)	13 (81.3%)	98 (86.7%)	
AGENT	1 (0.8%)	0 (0.0%)	1 (0.9%)	
Swide	14 (10.9%)	2 (12.5%)	12 (10.6%)	
SeQuent Please and AGENT	1 (0.8%)	1 (6.3%)	0 (0.0%)	
SeQuent Please and Swide	2 (1.6%)	0 (0.0%)	2 (1.8%)	
Number of DCBs used, *n* (%)				0.164
1 DCB	95 (74.2%)	10 (68.8%)	84 (75.0%)	
2 DCB	28 (21.9%)	3 (18.8%)	25 (22.3%)	
3 DCB	5 (3.9%)	2 (12.5%)	3 (2.7%)	
DCB diameter (mm)	2.80 ± 0.42	2.70 ± 0.35	2.82 ± 0.43	0.281
DCB length (mm)	31.7 ± 15.7	35.1 ± 19.17	31.1 ± 14.98	0.424
Long diffuse lesion (>60 mm), *n* (%)	12 (7.8%)	3 (18.8%)	7 (6.2%)	0.109
DCB Inflation time	67.3 ± 17.8	76.0 ± 22.3	65.6 ± 16.5	0.247
DCB maximal inflation pressure, atm	8.7 ± 3.0	8.0 ± 2.0	8.8 ± 3.2	0.612
Non-flow limiting dissection after DCB	8 (6.2%)	1 (6.3%)	7 (6.2%)	1.000
DCB to reference vessel ratio	0.95 ± 0.11	0.96 ± 0.13	0.96 ± 0.11	0.330

*p*-value represents comparison between TLR and non-TLR groups. DCB, drug coated balloon; DES, drug eluting stent; TLR, target lesion revascularization.

Procedural and lesion characteristics demonstrated several notable distinctions between groups. SeQuent Please was the predominant DCB used in this study, accounting for 86.0% of all treated lesions. Calcified lesions were significantly more frequent in the TLR cohort compared with the non-TLR group (93.8% vs. 54.9%; *p* = 0.003). Long lesion (>60 mm) and DCB-only strategy were also more frequently observed among lesions in TLR groups. Conversely, target lesion location, reference vessel diameter, choice of predilation balloon, type of DCB employed, and the use of atherectomy did not differ significantly between groups.

Lesion preparation included high rates of intravascular imaging guidance, with 88 lesions (68.2%) guided by IVUS, 14 (10.9%) by OCT, and 2 (1.6%) by both modalities. IVI-guided angioplasty was more frequently performed in lesions with larger vessel caliber (78.3%), calcification (69.8%), and bifurcation lesions (24.5%) compared with those treated without imaging (Table 5). However, IVI utilization was not independently associated with a lower TLR or MACE rates in this cohort.

**Table 5:** Lesion characteristics based on use of intravascular imaging.

Lesion Characteristics	Imaging	Without Imaging	*p*-value
	(*n* = 106)	(*n* = 23)	
Reference vessel diameter, *n* (%)			<0.001*
Small vessel (<2,75 mm)	23 (21.7%)	16 (69.6%)	
Non-small vessel (≥2,75 mm)	83 (78.3%)	7 (30.4%)	
Type of lesion			
Calcified Lesion, *n* (%)	74 (69.8%)	3 (13.0%)	<0.001*
Chronic Total Occlusion, *n* (%)	11 (10.4%)	5 (21.7%)	0.162
Multivessel Disease, *n* (%)	93 (87.7%)	20 (87.0%)	0.918
Bifurcation, *n* (%)	26 (24.5%)	0 (0.0%)	0.004*
Ostial, *n* (%)	32 (30.2%)	6 (26.1%)	0.696
Long diffuse (≥60 mm), *n* (%)	9 (8.5%)	1 (4.3%)	0.690
TLR	14 (13.5%)	2 (8.0%)	0.736
Clinical Outcome (patient-level)	Imaging	Without Imaging	*p*-value
	(*n* = 92)	(*n* = 20)	
MACE	12 (21.7%)	2 (14.3%)	0.709

Categorical data are presented as counts (%) and were analyzed using the chi-square test or Fisher's exact test, as appropriate.

* Indicates statistical significance (*p* value ≤ 0.05).

In IVUS-guided lesions (*n* = 90), plaque morphology and IVUS-derived calcium score distributions differed numerically between groups (Table 6). Lesions that developed TLR demonstrated a higher proportion of calcified and fibrocalcified plaques, although this trend did not reach statistical significance (*p* = 0.062). IVUS calcium score distribution was comparable between the TLR and non-TLR groups (*p* = 0.408).

**Table 6:** Analysis of lesion characteristics based on IVUS.

Characteristics (Lesion-based)	All Lesions	TLR	Non-TLR	*p*-value
	(*n* = 90)	(*n* = 12)	(*n* = 78)	
Reference Diameter, median (IQR)	3.17 (0.77)	3.01 (0.71)	3.2 (0.82)	0.950
Plaque Morphology, *n* (%)				0.062
Calcified	14 (15.6%)	4 (33.3%)	10 (12.8%)	
Fibrocalcified	49 (54.4%)	8 (66.7%)	41 (52.6%)	
Fibrotic	18 (20.0%)	0 (0.0%)	18 (23.1%)	
Fibrolipidic	9 (10,0%)	0 (0.0%)	9 (11.5%)	
IVUS Calcium Score, *n* (%)	All Lesion	TLR	Non-TLR	*p*-value
	(*n* = 63)	(*n* = 12)	(*n* = 51)	0.408
0	17 (27.0%)	2 (16.7%)	15 (29.4%)
1	19 (30.2%)	3 (25.0%)	16 (31.4%)	
2	15 (23.8%)	4 (33.3%)	11 (21.6%)	
3	8 (12.7%)	1 (8.3%)	7 (13.7%)	
4	4 (6.3%)	2 (16.7%)	2 (3.9%)	
Characteristics (Patient-based)	All Patient	MACE	No MACE	*p*-value
	(*n* = 78)	(*n* = 10)	(*n* = 68)	0.098
Calcified	11 (14.1%)	3 (30.0%)	8 (11.8%)	
Fibrocalcified	42 (53.8%)	7 (70.0%)	35 (51.5%)	
Fibrotic	17 (21.8%)	0 (0.0%)	17 (25.0%)	
Fibrolipidic	8 (10.3%)	0 (0.0%)	8 (11.8%)	
IVUS Calcium Score, *n* (%)	All Patient	MACE	No. MACE	*p*-value
	(*n* = 53)	(*n* = 10)	(*n* = 43)	0.617
0	14 (26.4%)	1 (10.0%)	13 (30.2%)	
1	17 (32.1%)	3 (30.0%)	14 (32.6%)	
2	13 (24.5%)	3 (30.0%)	10 (23.3%)	
3	6 (11.3%)	2 (20.0%)	4 (9.3%)	
4	3 (5.7%)	1 (10.0%)	2 (4.7%)	

*p* values for categorical variables were calculated using Fisher's exact test with Monte Carlo simulation due to sparse cell counts and should be interpreted as exploratory. Continuous variables were compared using the Mann–Whitney *U* test.

In the OCT-guided subcohort (*n* = 16), two lesions developed TLR. Fibrocalcified plaque was the predominant morphology overall (50.0%) and was present in both TLR cases, whereas calcified, fibrotic, and fibrolipidic plaques were observed in the non-TLR group. Assessment of calcium burden by OCT demonstrated a high prevalence of moderate to severe calcification, with OCT calcium scores of 3 or 4 observed in 69.3% of lesions. Both TLR events occurred in lesions with higher calcium scores (score 3 and 4). Given the small sample size and limited number of clinical events, these findings are presented descriptively without formal statistical comparison (Table 7).

**Table 7:** Analysis of lesion characteristics based on OCT.

Characteristics	All Lesions	TLR	Non-TLR
	(*n* = 16)	(*n* = 2)	(*n* = 14)
Reference Diameter, median (IQR)	2.77, (0.44)	2.65 (N/A)	2.79, (0.48)
Plaque Morphology, *n* (%)			
Calcified	5 (31.3%)	0 (0.0%)	5 (35.7%)
Fibrocalcified	8 (50.0%)	2 (100.0%)	6 (42.9%)
Fibrotic	2 (12.5%)	0 (0.0%)	2 (14.3%)
Fibrolipidic	1 (6.3%)	0 (0.0%)	1 (7.1%)
OCT calcium score, *n* (%)	All Lesion	TLR	Non-TLR
	(*n* = 13)	(*n* = 2)	(*n* = 11)
0	0 (0.0%)	0 (0.0%)	0 (0.0%)
1	0 (0.0%)	0 (0.0%)	0 (0.0%)
2	4 (30.8%)	0 (0.0%)	4 (36.4%)
3	3 (23.1%)	1 (50.0%)	2 (18.2%)
4	6 (46.2%)	1 (50.0%)	5 (45.5%)
Plaque Morphology, *n* (%)	All Patient	MACE	No. MACE
	(*n* = 16)	(*n* = 2)	(*n* = 14)
Calcified	5 (31.3%)	0 (0.0%)	5 (35.7%)
Fibrocalcified	8 (50.0%)	2 (100.0%)	6 (42.9%)
Fibrotic	2 (12.5%)	0 (0.0%)	2 (14.3%)
Fibrolipidic	1 (6.3%)	0 (0.0%)	1 (7.1%)
OCT calcium score, *n* (%)	All Patient	MACE	No MACE
	(*n* = 13)	(*n* = 2)	(*n* = 11)
0	0 (0.0%)	0 (0.0%)	0 (0.0%)
1	0 (0.0%)	0 (0.0%)	0 (0.0%)
2	4 (30.8%)	0 (0.0%)	4 (36.4%)
3	3 (23.1%)	1 (50.0%)	2 (18.2%)
4	6 (46.2%)	1 (50.0%)	5 (45.5%)

Due to the small sample size and limited number of clinical events in the OCT subgroup, statistical comparisons were not performed, and results are presented for descriptive purposes only.

In an exploratory subanalysis of severely calcified lesions (*n* = 40), atherectomy was performed in 15 lesions (37.5%), including rotational atherectomy in 7 lesions (17.5%) and orbital atherectomy in 8 lesions (20.0%). The distribution of atherectomy strategies did not differ significantly between lesions with and without subsequent target lesion revascularization. Notably, no TLR events were observed among lesions treated with orbital atherectomy; however, given the limited number of events, this finding should be interpreted cautiously. Detailed findings are presented in Table 8.

**Table 8:** Distribution of atherectomy strategies according to TLR Status in severely calcified lesions.

	All lesions	TLR	Non-TLR	*p*-value
	(*n* = 40)	(*n* = 9)	(*n* = 31)	
Atherectomy				0.234
No	25 (62.5%)	7 (77.8%)	18 (58.1%)	
Rotational	7 (17.5%)	2 (22.2%)	5 (16.1%)	
OAS	8 (20.0%)	0 (0.0%)	8 (25.8%)	

This subanalysis was exploratory in nature and not powered to detect differences between atherectomy modalities. Accordingly, comparisons should be interpreted as descriptive and hypothesis-generating.

Non–flow-limiting dissection following DCB angioplasty was observed in 8 lesions (6.2%), with no significant difference between groups (6.3% vs. 6.2%; *p* = 1.00).

### 3.3 Predictors of target lesion revascularization

Multivariable logistic regression identified calcified lesions (adjusted OR 9.93; 95% CI, 1.23–80.49; *p* = 0.032) as independent predictors of TLR (Table 9). In contrast, male sex, smoking, DCB-only approach, type of DCB used, and long lesion (≥60 mm) were not associated with TLR.

**Table 9:** Independent predictors of target lesion revascularization in multivariate analysis.

Variable	Unadjusted OR (95% CI)	*P* value	Adjusted OR (95% CI)	*P* value
Calcified Lesions	12.34 (1.58–96.60)	0.017*	9.93 (1.26–80.49)	0.032*
Sex, male	5.18 (0,66–40.95)	0.119	3.35 (0.32–34.68)	0.655
Smoking	3.69 (0.99–13.67)	0.050	2.99 (0.65–13.76)	0.160
Long Lesion (>60 mm)	3.49 (0.80–15.19)	0.095	3.49 (0.61–19.88)	0.158
DCB-only	2.82 (0.96–8.31)	0.061	2.94 (0.84–10.28)	0.091
SeQuent Please DCB	0.67 (0.17–2.63)	0.566	0.84 (0.07–10.67)	0.893
Swide DCB	1.19 (0.24–5.89)	0.831	1.68 (0.09–32.94)	0.733

Model statistics: Omnibus *χ*² = 21.33 (df = 6), *p* = 0.002; Nagelkerke R² = 0.289; Hosmer–Lemeshow *p* = 0.242.

OR, odds ratio; CI, confidence interval.

* Indicates statistical significance (*p* value ≤ 0.05).

No significant multicollinearity was detected among candidate predictors (all VIF < 2; highest condition index = 13.7). The overall model was statistically significant (Omnibus *χ*² = 21.33, df = 6, *p* = 0.002). Model calibration was adequate based on the Hosmer–Lemeshow test (*χ*² = 7.94, df = 6, *p* = 0.242), with a −2 log-likelihood of 75.39 and a Nagelkerke R² of 0.289.

### 3.4 Clinical outcomes

MACE was evaluated at the patient level (*n* = 112 patients). All patients completed at least 12 months of clinical follow up, during which MACE occurred in 14 patients (12.5%). No all-cause or cardiac deaths were observed within the first 12 months. With extended follow-up of up to 24 months (median follow-up duration, 17 months; IQR, 9 months), MACE occurred in 17 patients (15.18%). All-cause mortality was 0.8%, and no cardiac deaths were recorded. Detailed cumulative clinical outcomes are summarized in Table 10.

**Table 10:** Cumulative events of patient-based clinical outcomes (*n* = 112).

Clinical Outcomes, *n* (%)	≤30 days	≤6 months	≤12 months	≤18 months	≤24 months
All-cause death	0	0	0	1 (0.89%)	1 (0.89%)
Cardiac death	0	0	0	0	0
Non-cardiac death	0	0	0	1 (0.89%)	1 (0.89%)
Recurrent MI	0	0	1 (0.89%)	1 (0.89%)	3 (2.68%)
TLR	0	6 (5.36%)	12 (10.71%)	12 (10.71%)	12 (10.71%)
Rehospitalization	0	0	0	1 (0.89%)	2 (1.79%)
CVA	0	0	1 (0.89%)	1 (0.89%)	1 (0.89%)
MACE (composite)	0	6 (5.36%)	14 (12.5%)	15 (13.39%)	17 (15.18%)

Values are presented as cumulative event counts. Clinical outcomes including TLR are reported on a patient basis. Follow-up was non-uniform, with all patients followed for at least 12 months and a subset followed up to 24 months. CVA, cerebrovascular accident; TLR, target lesion revascularization; MACE, major adverse cardiovascular events (a composite of cardiac death, recurrent myocardial infarction, target lesion revascularization, unplanned rehospitalization, or cerebrovascular accident).

Intravascular imaging subanalyses demonstrated no significant association between plaque morphology or IVUS-derived calcium score and the occurrence of MACE. Although patients who experienced MACE showed a numerically higher proportion of calcified and fibrocalcified plaques, no statistically significant differences were observed for plaque morphology (*p* = 0.098) or IVUS calcium score (*p* = 0.617). Similarly, plaque morphology and OCT-derived calcium score distributions were comparable between patients with and without MACE. Detailed imaging findings are presented in Tables 6, 7.

**Figure 2:**
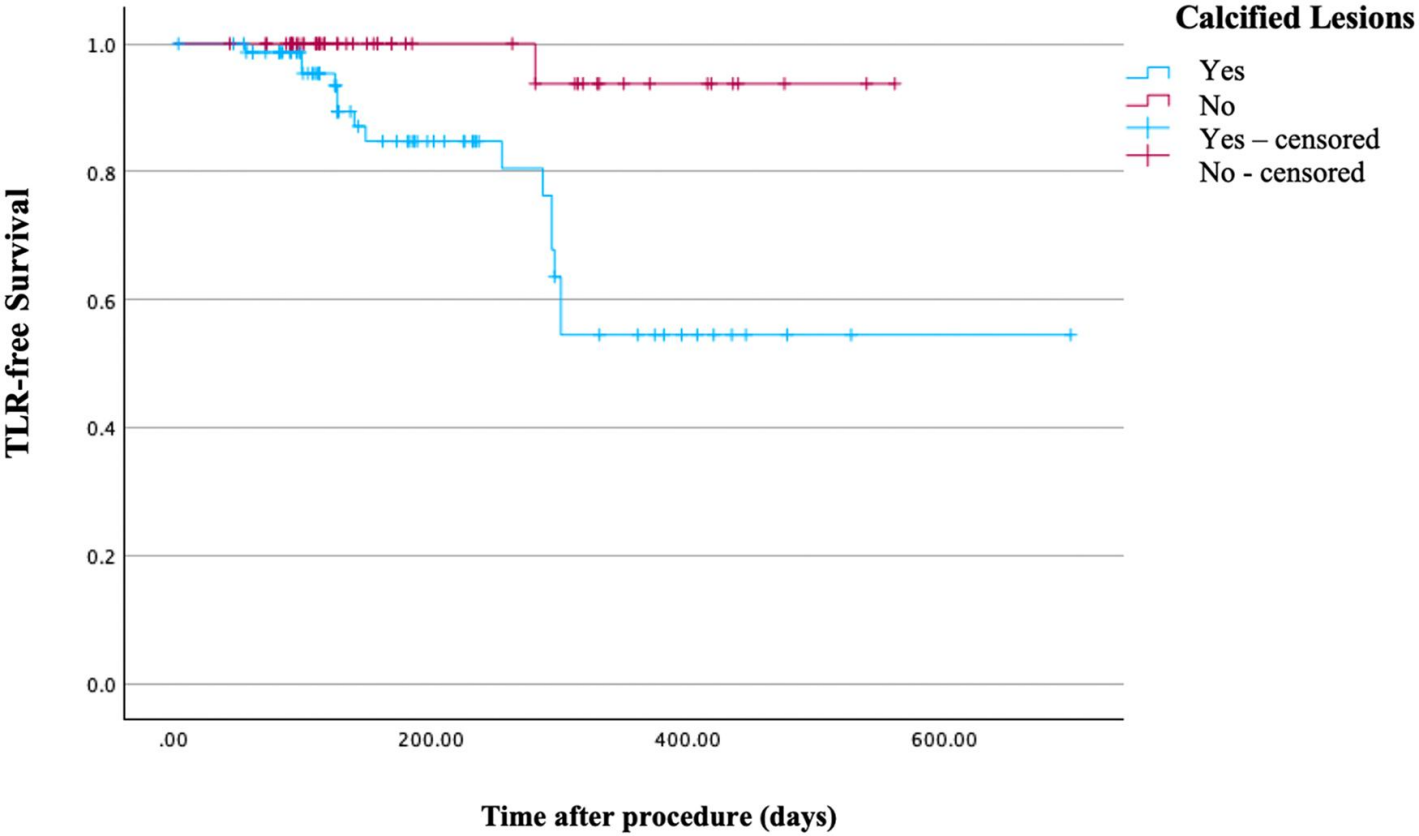
Kaplan–Meier curves for freedom from target lesion revascularization stratified by lesion calcification. Calcified lesions demonstrated significantly lower TLR-free survival compared with non-calcified lesions (log-rank *p* = 0.003).

**Figure 3:**
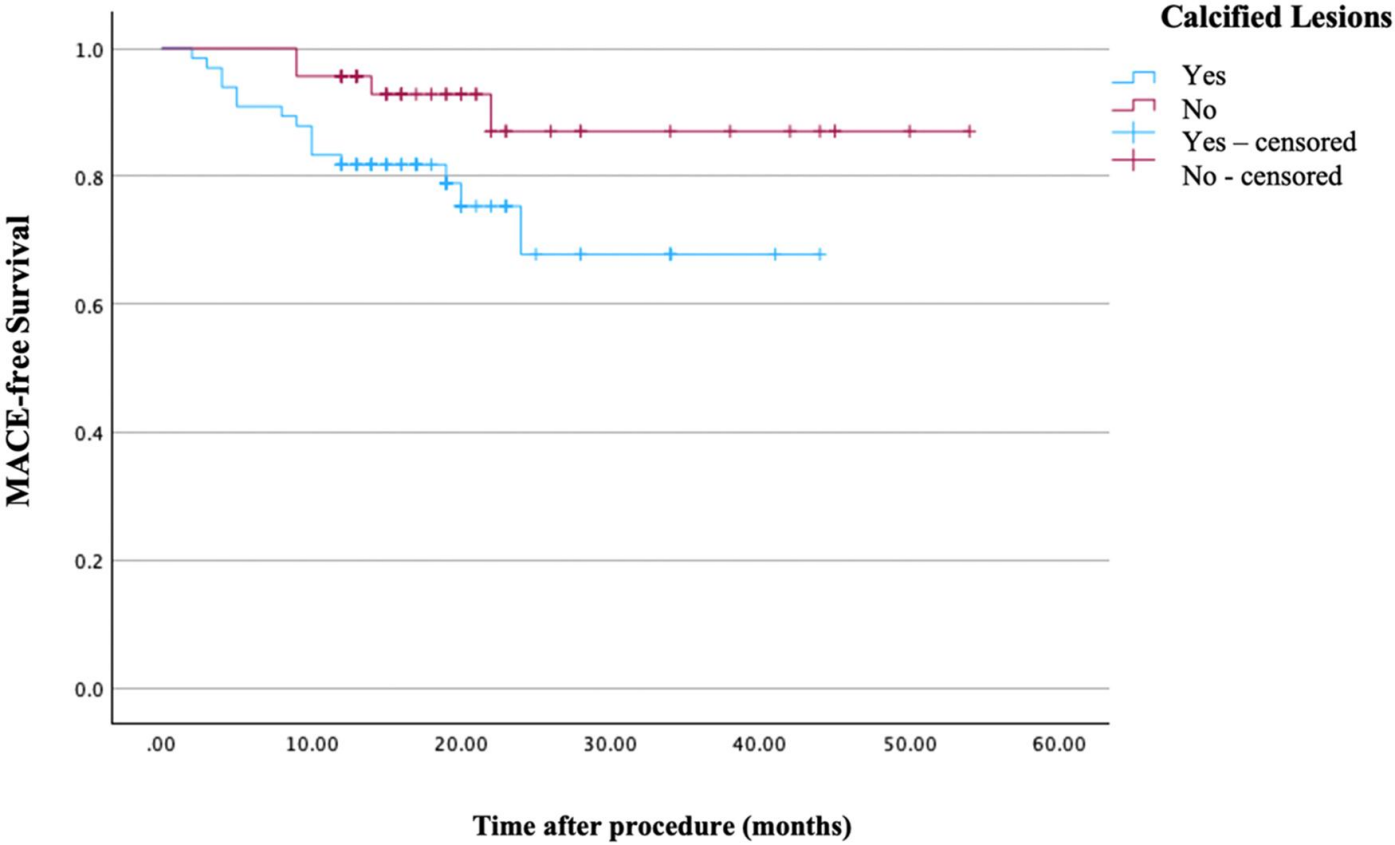
Kaplan–Meier curves for freedom from major adverse cardiovascular events stratified by lesion calcification. A significant difference in MACE-free survival was observed between groups (log-rank *p* = 0.040).

Kaplan–Meier analysis demonstrated significantly lower freedom from TLR and MACE in calcified lesions compared with non-calcified lesions (log-rank *p* = 0.003 for TLR and *p* = 0.040 for MACE; Figures 2, 3).

## 4 Discussion

**Figure 4:**
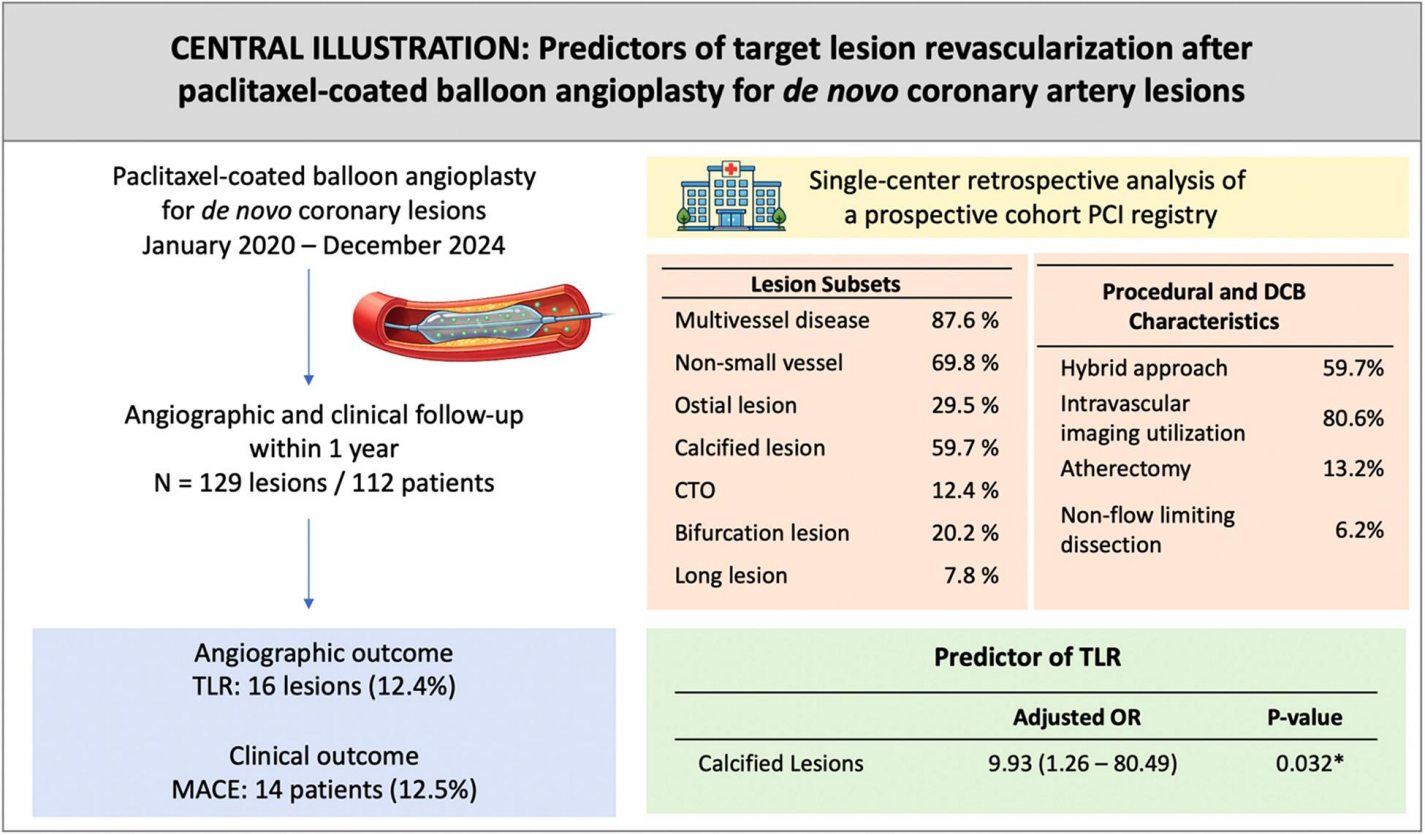
Central Illustration. Note: DCB, drug-coated balloon; PCI, percutaneous coronary intervention; CTO, chronic total occlusion; TLR, target lesion revascularization; MACE, major adverse cardiovascular events; OR, odds ratio.

This cohort study investigating DCB angioplasty in *de novo* CAD in various clinical settings provides valuable real-world evidence in a Southeast Asian population—a region that remains underrepresented in current cardiovascular research (11). The principal finding was calcified lesions independently predicted TLR (Figure 4). The 12-month TLR rate was 12.4%, modestly higher than rates reported in large multicenter DCB registries.

Compared with previous DCB registries, the TLR incidence in our study was higher. In a large-scale multicenter Korean registry, which included 1.184 *de novo* lesions, the 12-month TLR rate was notably low (1.7%). However, several notable differences in lesion and procedural characteristics should be considered when interpreting this finding, including markedly lower proportion of calcified lesions (5.8%), lower burden of multivessel disease (65.0%), and shorter mean DCB length (22.2 ± 5.4 mm) compared with our cohort (31.7 ± 15.7 mm), factors that likely contributed to the lower TLR rate (12).

In a prospensity-matched multicenter study from China, the three-year TLR incidence was significantly higher in calcified vs. non-calcified group (7.41% vs. 2.88%; OR, 2.64; 95% CI, 1.21–5.79; *p* = 0.020). Compared to our cohort, this study involved equal proportion of calcified and noncalcified group following prospensity matching, and shorter mean DCB length (2.70 ± 0.41 mm) (13). Likewise, Japan reported a 7.6% incidence of TLR in OCT-guided PCI registry, with severe calcification—characterized by a greater maximum calcium arc (median 215° vs. 104°)—was identified as a significant predictor of target lesion failure following DCB angioplasty. Compared to our study, this registry incorporated intravascular imaging in all lesions, and demonstrated higher utilization of coronary atherectomy and excimal laser (14).

Taken together, several factors likely contributed to the higher observed TLR rate in our cohort, including a greater prevalence of calcified lesions, the use of longer DCBs, lower utilization of coronary atherectomy, and incorporation of angiographic surveillance which may have increased detection of restenosis cases during scheduled follow-up angiography or staged procedures, irrespective of clinical symptoms. The adoption of a more liberal routine angiographic follow-up strategy at our center may have increased restenosis detection and reintervention rates. In contrast, favorable outcomes reported in some East Asian registries may also be partially attributable to lower thrombogenicity observed in this population, contributing further to inter-study variability (12).

Percutaneous coronary intervention of calcified coronary lesions is associated with increased short-term and long-term risk (8). In this study, calcified lesions independently predicted TLR after DCB angioplasty. Mechanistically, vascular calcium acts as a diffusion-limiting barrier, impeding paclitaxel penetration and retention within the medial smooth-muscle layer and thereby attenuating its antiproliferative efficacy (15, 16). Suboptimal calcium modification prior to intervention and limited utilization of intravascular imaging in some calcified lesions may have further contributed to restenosis risk. Nevertheless, a DCB-only approach remains a viable option in selected calcified *de novo* lesions, particularly when deployment or expansion of a metallic DES is unlikely to be feasible or effective (2).

Stent implantation in calcified lesions remains technically challenging, with impaired stent delivery and suboptimal stent expansion leading to unfavorable long-term outcomes (17, 18). In heavily calcified lesions, stent polymer and drug coating could be damaged, resulting in stent failure such as thrombosis or restenosis (17). Multiple studies have demonstrated less favorable outcomes with TLR rates of between 6% and 8% at 1-year and up to 12%–14% at 2 years (19) Management of restenosis in calcified DES segments presents unique challenges (20). DCB angioplasty has gained increasing interest as a “leave nothing behind” strategy that eliminates the risk of stent under-expansion due to calcium (19). However, DCB use in calcified coronary disease results in higher target lesion failure than non-calcified lesions (14).

The 2024 SCAI Consensus Statement emphasizes the importance of intravascular imaging (IVUS or OCT) to guide calcium modification strategies, such as atherectomy or lithotripsy, prior to intervention. Although SCAI does not issue a class recommendation for DCB in calcified *de novo* lesions, it emphasizes imaging-guided calcium modification to optimize outcomes (8). In severely calcified lesions, rotational atherectomy (RA) or orbital atherectomy (OAS) followed by DCB yielded TLR and MACCE rates comparable to RA or OAS followed by DES implantation, suggesting that adequate lesion preparation may mitigate restenosis risk (21, 22).

Atherectomy-based plaque modification, particularly techniques capable of achieving circumferential calcium fracture, may theoretically enhance paclitaxel uptake and reduce restenosis (8). In the subanalysis of atherectomy strategies in severely calcified lesions (Table 8), atherectomy guided with intravascular imaging was performed in 15 of 40 lesions (37.5%), including RA in 7 lesions (17.5%) and OAS in 8 lesions (20.0%). Excimal laser coronay atherectomy and intravascular lithotripsy were not available in our center during the study period.

Although no statistically significant association between atherectomy strategy and TLR was observed (*p* = 0.234), several observed findings are noteworthy. No TLR observed among lesions treated with orbital atherectomy, TLR rates were comparable in lesions treated with rotational atherectomy, and higher proportion of TLR occurred in severely calcified lesions that did not undergo atherectomy (77.8% vs. 58.1%). Orbital atherectomy works through centrifugal force and surface friction, creating elliptical orbits that change the compliance of calciﬁed vessels by altering the depth of calcium and creating microfractures (8). Although calcified plaque limited intravascular drug delivery, controlled OAS resulted in greater drug permeability and enhance drug delivery to diseased calcified arteries (23).

Our findings suggest that more aggressive calcium-modification strategies may potentially influence restenosis risk following DCB angioplasty in severely calcified lesions. Given the limited sample size and low event numbers in this subgroup, comparisons regarding atherectomy strategies were descriptive and hypothesis-generating. Larger, adequately powered studies are warranted to clarify the role of atherectomy in optimizing outcomes of DCB-based interventions in severely calcified lesions.

The hybrid DES/DCB approach has been evaluated in long *de novo* lesions and diffuse coronary artery disease. In this approach, a DES was implanted in the proximal lesion, and DCB angioplasty was performed in the distal lesion, thereby reducing total stent length, which is beneficial for lower restenosis rates (3). The hybrid approach dominated lesion revascularization in our study (59.7%), reflecting operator preference due to high prevalence of multivessel CAD (87,6%), non-small vessel disease (69.8%), and intervention of long lesion and bifurcation lesions for which contemporary guidelines favors DES implantation (1, 4, 5). In subgroup analysis, TLR occurred in 19.2% of lesions treated with a DCB-only strategy compared with 7.8% in the hybrid DES/DCB group. In unadjusted logistic regression, DCB-only treatment was associated with higher odds of TLR (OR 2.82, 95% CI 0.96–8.31; *p* = 0.061); however, this did not reach statistical significance. After multivariable adjustment, the procedural strategy was not independently associated with TLR.

In multivessel CAD, Shin et al. reported that the DCB-based treatment approach significantly reduced stent burden and was associated with lower rate of MACE than the DES-only treatment by reducing stent-related events (24). Real world data similarly support selective DES use with DCB in bifurcation, diffuse, and calcified disease, leveraging DCB's “leave-nothing-behind” advantage while providing scaffolding where necessary, and has the potential as alternative strategy to treat more complex lesions than DCB-only strategy (25–27). Collectively, our findings and prior evidence support a tailored hybrid approach in complex *de novo* CAD (14, 24).

Intravascular imaging was frequently employed, primarily in larger vessel, bifurcation, and calcified lesions. Higher proportion of non-small vessels and calcified lesions observed in the imaging-guided group should be interpreted cautiously, as EEL-based intravascular measurements tend to yield larger reference diameters than angiographic estimates, and intravascular imaging is more sensitive for detecting and characterizing calcification than angiography alone. Neither vessel size nor bifurcation lesions were associated with an increased risk of TLR, underscoring the procedural safety of DCB angioplasty in this setting when guided by intravascular imaging.

Although intravascular imaging (IVI) was used in approximately 80% of lesions, it was not independently associated with reduced TLR. This finding likely reflects confounding by indication, as IVI was preferentially utilized in more complex lesions, which inherently carry a higher restenosis risk. Therefore, absence of an independent protective effect should not be interpreted as lack of clinical benefit of IVI, but rather as a limitation inherent to the observational design. Incorporation of intravascular imaging in DCB angioplasty and managing calcified lesion is reasonable to further define calcium phenotypes, carry out quantitative analysis of calcium plaque, and determine the need and optimal calcium modification strategies (8, 14, 28).

In the subanalysis of IVUS and OCT findings, although higher-grade IVUS and OCT calcium scores appeared more frequent among TLR and MACE cases, these observations are exploratory and hypothesis-generating, as the study was underpowered for definitive subgroup analyses. Neither plaque morphology nor IVUS calcium score was independently associated with TLR or MACE. Although lesions that developed TLR more frequently exhibited calcified and fibrocalcified plaque phenotypes, these differences did not reach statistical significance, likely reflecting limited statistical power and sparse event counts.

In our center, operators were not mandated to use the same DCB vendor when more than one balloon was required for a lesion (Table 4). Nonetheless, most lesions (86%) were treated with balloons from the single manufacturer, which limit the study's ability to compare outcomes across different DCB platforms or drug-dose densities. Although lesions treated with three DCBs exhibited a higher incidence of TLR, this observation likely reflects underlying lesion complexity—such as long, diffuse, or multi-segment disease—rather than an adverse effect of balloon number itself. Notably, the two cases (3 lesions) that developed TLR after treatment with three DCBs involved long lesions (>60 mm) with multi-segment DCB application (proximal-to-distal LAD and mid-to-distal LAD) in the setting of severe coronary calcification, as confirmed by intravascular ultrasound (calcium scores of 4 and 3, respectively) Given the small number of lesions requiring three DCBs, no definitive conclusions regarding the impact of three DCB per lesion on TLR can be drawn. Current consensus guidance focuses on adequate lesion preparation and complete drug coverage (prepared lesion plus ∼2 mm margin), not limiting the numeric count of DCB (29).

Our study demonstrated no significant difference in the frequency of coronary risk factors at the index PCI between the two groups. Similarly, several published studies also reported no independent association between baseline coronary risk factors and TLR (14, 26).

The 1-year MACE rate observed in the present study (12.5%) was modestly higher than that reported in prior studies. It is important to note that MACE was evaluated at the patient level, whereas TLR was analyzed at the lesion level. Lee et al. reported a 12-month MACE rate of 7.5%; however, their cohort included a very low proportion of calcified lesions (12). In contrast, Shan et al. demonstrated a numerically higher incidence of MACE in patients with coronary artery calcification compared with those without calcification during 3-year follow-up (12.35% vs. 7.82%; OR 1.665; 95% CI, 0.951–2.916), although this difference did not reach statistical significance (13). Reported MACE rates from randomized controlled trials evaluating DCB angioplasty in *de novo* lesions also vary widely. Trials involving small-vessel disease reported 9–12-month MACE rates ranging from 8% to 35.7%, whereas studies in larger coronary vessels demonstrated lower rates of 0%–9.4% at similar follow-up intervals (29).

In major clinical trials, restenosis following DCB angioplasty is typically evaluated angiographically at 6–9 months, corresponding to the anticipated time course of neointimal proliferation. Clinical follow-up in these studies generally extends from 6 months up to 2 years to capture subsequent adverse events (7, 12, 14, 22, 30–33). Accordingly, the observed 12.4% TLR rate should be interpreted within the context of intermediate-term angiographic surveillance. Longer-term clinical follow-up would provide a more comprehensive assessment of treatment durability and late event patterns.

The present study results support the feasibility of DCB angioplasty across a broad spectrum of *de novo* coronary lesions—including multivessel, bifurcation, CTO, and non-small-vessel disease—provided that meticulous lesion preparation and IVI guidance are ensured. Although intravascular imaging offers important procedural advantages, such as plaque characterization, accurate vessel sizing, and optimization of lesion preparation, its clinical impact on TLR could not be demonstrated in this cohort. This observation likely reflects preferential use in more complex lesions and the inherent limitations of observational analyses. Consequently, the findings of this study should not be interpreted as diminishing the value of intravascular imaging, but rather highlight the challenges of assessing its impact on clinical outcomes within observational study designs.

Several limitations merit consideration. First, procedural decisions—including DCB diameter and length—were left to operator discretion, introducing potential selection and performance bias. Utilization of intravascular imaging was not randomized and preferentially utilized in more complex lesion, therefore residual confounding cannot be excluded. Second, the sample size was modest, which may limit statistical power and the precision of effect estimates; therefore, the results should be considered exploratory and hypothesis-generating. Third, intravascular imaging was not uniformly performed in all lesions. Vessel sizing and calcium detection were assessed using different modalities, and primarily affect the descriptive comparison between groups. Reliance on fluoroscopy—whose sensitivity for detecting target-lesion calcium are approximately 50% when compared with intravascular imaging—limits calcium detection and characterization (8). In the non-imaging guided group, quantitative coronary angiography was not performed systematically, and classification into small vs. non-small vessels relied on operator visual estimation and the selected balloon diameter. Fourth, the use of different DCB types—although uncommon—may introduce heterogeneity due to variations in paclitaxel formulation, dose density, and coating characteristics. Fifth, the relatively short median angiographic follow-up may underestimate late TLR events and limits assessment of long-term durability. Sixth, as this cohort represents an Indonesian population, extrapolation to other ethnic and healthcare settings should be approached with caution.

## 5 Conclusion

In this real-world, single-center cohort of patients undergoing DCB angioplasty for *de novo* CAD, calcified lesions emerged as independent predictors of TLR. This observation underscores the potential impact of calcium burden on drug delivery and procedural efficacy in DCB-based interventions. Larger multicenter, prospective studies are needed to confirm these observation and to optimize lesion-specific DCB strategies.

## Data Availability

The raw data supporting the conclusions of this article will be made available by the authors, without undue reservation.
