# Supplementary material for: Predictors of target lesion revascularization after paclitaxel-coated balloon angioplasty for de novo coronary artery lesions
Source: Front Cardiovasc Med. 2026 Mar 24;13:1724573. doi: 10.3389/fcvm.2026.1724573 (PMC13055610; doi:10.3389/fcvm.2026.1724573)
Supplement: Supplementary file 1 [file Table1.docx]

Supplementary Material

**Supplementary Table 1. Multicollinearity** **Assessment of Candidate Predictors**

|  | **Tolerance** | **VIF** |
| --- | --- | --- |
| Calcified Lesions | 0.920 | 1.087 |
| Sex, male | 0.676 | 1.480 |
| Smoking | 0.648 | 1.544 |
| Long Lesion (>60 mm) | 0.966 | 1.035 |
| DCB-only | 0.929 | 1.076 |
| Type of DCB used | 0.923 | 1.083 |

Note: VIF= variance inflation factors. VIF >5 was considered indicative of significant multicollinearity. The maximum observed VIF was 1.544.
